# Application of Near-Infrared Spectroscopy to Monitor Perfusion During Extracorporeal Membrane Oxygenation After Pediatric Heart Surgery

**DOI:** 10.3389/fmed.2021.762731

**Published:** 2021-11-22

**Authors:** Mingjie Zhang, Yinyu Yang, Xi Chen, Yixiao Song, Limin Zhu, Xiaolei Gong, Haibo Zhang, Zhuoming Xu

**Affiliations:** Department of Thoracic and Cardiovascular Surgery, Shanghai Children's Medical Center, School of Medicine, Shanghai Jiao Tong University, Shanghai, China

**Keywords:** ECMO perfusion, NIRS, pediatric, heart surgery, mortality

## Abstract

**Objective:** Venoarterial extracorporeal membrane oxygenation is an effective mechanical circulatory support that is used to rescue critically ill patients after congenital heart surgery. As there was still no recommended guideline for monitoring parameters during extracorporeal membrane oxygenation (ECMO), this study aimed to investigate the role of near-infrared spectroscopy (NIRS) in the early period of venoarterial (VA)-ECMO.

**Method:** This study enrolled patients with NIRS monitoring during ECMO after pediatric cardiac surgery at Shanghai Children's Medical Center (2018–2020). The information obtained from the retrospective, the observational dataset included the demographic information, diagnoses, baseline characteristics, procedural details, ECMO data, monitoring data, in-hospital mortality, and complications of the patients.

**Results:** The overall mortality rate was 43.6%. Lactate was significantly higher in non-survivors compared to survivors at 12 h (11.25 ± 7.26 vs. 6.96 ± 5.95 mmol/l, *p* = 0.022) and 48 h [2.2 (0.7, 20) vs. 1.4 (0.7, 5.8) mmol/l, *p* = 0.008] after initiation of ECMO. The cranial regional oxygen saturation (CrSO_2_) was significantly higher in survivors compared to non-survivors at 24 h (62.5 ± 14.61 vs. 52.05 ± 13.98%, *p* = 0.028), 36 h (64.04 ± 14.12 vs. 51.27 ± 15.65%, *p* = 0.005), and 48 h (65.32 ± 11.51 vs. 55.00 ± 14.18%, *p* = 0.008). Multivariate logistics regression analysis of the hemodynamic and laboratory parameters revealed that the CrSO_2_ at 36 h (OR = 0.945, *p* = 0.049) and 48 h (OR = 0.919, *p* = 0.032) was related to mortality. The use of continuous renal replacement therapy (OR = 14.940, *p* = 0.039) was also related to mortality. The optimal cutoff values for CrSO_2_ for predicting mortality after weaning off ECMO at 36 and 48 h were 57% (sensitivity: 61.5%, specificity: 80%) and 56% (sensitivity: 76.9%, specificity: 70%), respectively. The risk of mortality was higher among patients with a CrSO_2_(36h) < 57% (*p* = 0.028) by Kaplan-Meier analysis.

**Conclusion:** Near-infrared spectroscopy may be a useful tool for monitoring the hemodynamic stability during the early period of ECMO, while CrSO_2_ can predict the in-hospital mortality after ECMO.

## Introduction

Extracorporeal membrane oxygenation is an effective method for the management of refractory cardiogenic shock (1). It is a life-saving procedure in the event of failure of other conventional therapies. Recent advancements in technology have expanded its applications to more complex diseases, including congenital heart disease (CHD) in children. Extracorporeal membrane oxygenation (ECMO) has been used in increasingly complicated cases of pediatric CHD during the early postoperative risk stage after open heart surgery in recent years. It was reported that up to 2–5% of all children undergoing cardiac surgery require mechanical cardiac support with ECMO during the postoperative period (2). Unlike adults, the body weight and blood volume vary over a wide range in children. Therefore, the clinical management of ECMO presents a huge challenge in children.

A variety of hemodynamic monitoring methods, including the pulse contour cardiac output and other invasive monitoring modalities, cannot be applied during the period of ECMO assistance. In 1977, Jobsis first used near-infrared spectroscopy (NIRS) for monitoring cerebral oxygen levels (3). The recent technological advancement in optical instruments and improvement in light propagation in tissues has facilitated the use of multisite NIRS technology for noninvasive and continuous monitoring of oxygen saturation in the brain and body tissue in clinical practice to facilitate real-time monitoring of blood and oxygen supply to the organs (4–6). Traditional monitoring parameters, such as blood pressure, cannot adequately reflect the oxygenation state of the brain and abdominal microcirculation, especially for the non-pulsatile blood flow generated by ECMO. The abnormalities in systemic oxygen balance can be detected through the monitoring of systemic venous oxygen saturation (SvO_2_), lactic acid, and noninvasive multisite NIRS (7). But the blood gas tests require frequent blood sampling which would cause anemia and cannot realize the real time. Thus, this study aimed to study the noninvasive monitoring parameters during ECMO after pediatric heart surgery related to organ perfusion and mortality and explore the clinical significance of multi-channel NIRS monitoring during the period of ECMO assistance.

## Materials and Methods

### Study Population

This retrospective study included children who underwent cardiac surgery between January 1, 2018, and December 31, 2020, and was approved by the medical ethics committee of Shanghai Children's Medical Center, School of Medicine, Shanghai Jiaotong University. The cerebral and abdominal oxygenation monitoring data were available for 56 of 124 patients who underwent venoarterial (V-A) ECMO. One patient was found with congenital hypertrophic cardiomyopathy with NIRS monitoring and was excluded. Therefore, a final total of 55 cases were included in this study. The participants were under 18 years of age. Any death occurring before hospital discharge was designated as death after weaning off ECMO. Congenital heart surgery was defined as any surgical procedure for a cardiac defect with or without cardiopulmonary bypass (CPB). ECMO was implemented immediately after surgery in the operating room, after cardiopulmonary resuscitation in the cardiac intensive care unit (CICU), or selectively due to circulatory instability in the CICU.

### Data Collection

The information obtained from the retrospective, observational dataset included the demographic information, diagnoses, baseline characteristics, procedural details, ECMO data, monitoring data, in-hospital mortality, and complications of patients. The CICU monitoring indices included blood pressure, central venous pressure (CVP), blood gas, and lactic acid.

Near-infrared spectroscopy monitoring (INVOS5100C, Covidien, USA) was conducted by affixing the head electrode approximately 1 cm above the eyebrow arch on the left or right side to measure the cranial regional oxygen saturation (CrSO_2_). The abdominal electrode was generally attached above or below the umbilicus to obtain the mesenteric regional oxygen saturation (MrSO_2_). The electrodes are used according to body weight, that is, <5, 5–40, and ≥40 kg.

### ECMO Management

Extracorporeal membrane oxygenation was implanted by a central cannulation way, and a left atrium drainage tube was placed in patients with insufficient intracardial shunting. Arterial blood pressure was maintained within the normal range of mean arterial pressure for different age groups. The pump inlet pressure exceeded −20 mmHg, and the outlet pressure was within 200 mmHg. The activated partial thromboplastin time (APTT), activated clotting time (ACT), and anti-Xa were monitored for anticoagulation. The target ACT value was 140–180 s, and the standard APTT and anti-Xa values were 40–80 s and 0.3–0.8 IU/ml, respectively. The ventilator was set to the pressure-regulated volume control mode with a positive end-expiratory pressure of 10 mmHg, tidal volume of 6–8 ml/kg, and respiratory rate of 10–12 bpm. Enteral nutrition was supplied to patients without obvious contraindications such as gastrointestinal bleeding, storage, or MrSO_2_ <35%. Diuretics were administered as soon as the ECMO flow was stabilized to prevent fluid overload.

### Statistical Analysis

All data were analyzed using SPSS 22.0 (IBM, Armonk, NY, USA). Data with normal distribution were presented as the mean ± SD. Abnormally distributed values were presented as the median and range (minim, max). The medians of the two groups were compared using the Mann-Whitney U test. Categorical data were represented as frequencies and percentages and were evaluated using the chi-squared test. Multivariable logistics regression models explored meaningful variables to predict mortality. The receiver operating characteristic (ROC) was used to determine the cut-off value of the monitoring index to distinguish between survivors and non-survivors. Kaplan-Meier analysis was used to analyze the survival between different subgroups according to the CrSO_2_. *P* < 0.05 was considered statistically significant.

## Results

Among the 55 patients, there were 16 types of CHD (Table 1). The first three types were transposition of the great arteries (TGA) (*n* = 10, 18.1%), pulmonary atresia (PA) (*n* = 6, 10.9%), and double outlet right ventricle (*n* = 6, 10.9%). Although there were some simple cases of CHD, such as ventricular septum defect, most of these patients had heart dysfunction or extracardiac problems before the procedure. Palliative surgery was performed in 22% of patients, including three patients with Ebstein's malformation, two patients with TGA, two with Tetralogy of Fallot, one with PA with intact ventricular septal, one with PA with VSD, and one patient with a single ventricle.

**Table 1:** Diagnosis of patients with extracorporeal membrane oxygenation (ECMO).

**Diagnosis**	**Number**
Transposition of great arteries	10
Aortic valve stenosis	7
Pulmonary atresia	6
Double outlet right ventricle	6
Ventricular septum defect	5
Interruption of the aortic arch	3
Ebstein's malformation	3
Mitral stenosis	3
Patent ductus arteriosus	3
Coarctation of the aorta	3
Anomalous left coronary artery from the pulmonary artery	2
Tetralogy of Fallot	1
Single ventricle	1
Total anomalous of pulmonary venous connection	1
Complete atrioventricular canal	1

Twenty-four patients died and the overall mortality was 43.6%. The causes of the death included heart failure (*n* = 7), multiple organ dysfunction (*n* = 6), residual anatomy (*n* = 5), pulmonary hypertension (*n* = 3), brain death (*n* = 2), and sepsis (*n* = 1).

No significant differences were observed between the age, sex, CPB time, and aortic cross-clamp (ACC) time of the survivors and non-survivors (Table 2). Significant differences were not observed in the number of patients who underwent different ECMO protocols, i.e., after cardiopulmonary resuscitation (ECPR) (*p* = 0.214) or in the operating room (OR) (*p* = 0.595). There was no significant difference in proportion between cyanotic CHD and non-cyanotic CHD (*p* = 0.551). The ECMO flow was higher among the neonatal non-survivors than that in the survivors (156.73 ± 8.71 vs. 124.48 ± 10.10 ml/kg/min, *p* = 0.001). But there is no significant difference between the neonates and infants in the CrSO_2_ (68.23 ± 11.40 vs. 64.93 ± 8.96%, *p* = 0.402). There were more patients who received continuous renal replacement therapies (CRRT) (*p* = 0.007). The duration of ECMO was longer in the non-survivor group (*p* = 0.033). The time required to achieve pulse pressure >10 mmHg was shorter in the survivor group than that in the non-survivor group (38.18 ± 28.95 vs. 59.44 ± 43.10 h, *p* = 0.044).

**Table 2:** Comparison of the clinical characteristics between survivors and non-survivors.

	**Total (*N* = 55)**	**Survivors (*N* = 31)**	**Non-survivors (*N* = 24)**	**P**
Age (months)	3 (0.03, 189.5)	3 (0.03, 189.5)	2.72 (0.17, 171.33)	
Sex (Female/Male)	21/34	11/20	10/14	0.640
CPB (min)	202.14 ± 135.55	199.86 ± 131.08	205.14 ± 144.28	0.892
ACC (min)	90.96 ± 60.11	80.37 ± 51.58	106.00 ± 69.17	0.157
ECPR	18	8	10	0.214
OR	23	12	11	0.595
Cyanotic/non-cyanotic	30/25	18/13	12/12	0.551
CRRT	8	1	7	0.007^*^
ECMO duration (h)	112.20 ± 73.44	91.74 ± 40.12	138.64 ± 96.27	0.033^*^
ECMO flow (ml/kg/min)				
Neonates (*n* = 10)	140.61 ± 19.18	124.48 ± 10.10	156.73 ± 8.71	0.001^*^
Infants /children (45)	113.34 ± 28.15	112.91 ± 25.75	109.20 ± 31.75	0.668
TΔp (h)	45.99 ± 35.90	38.18 ± 28.95	59.44 ± 43.10	0.044^*^

*CPB, cardiopulmonary bypass; ACC, arterial cross-clamp; ECPR, cardiopulmonary resuscitation; OR, operating room; CRRT, continuous renal replacement therapies; TΔp, time to achieve pulse pressure 10 mmHg*.

* *p < 0.05*.

**Figure 1:**
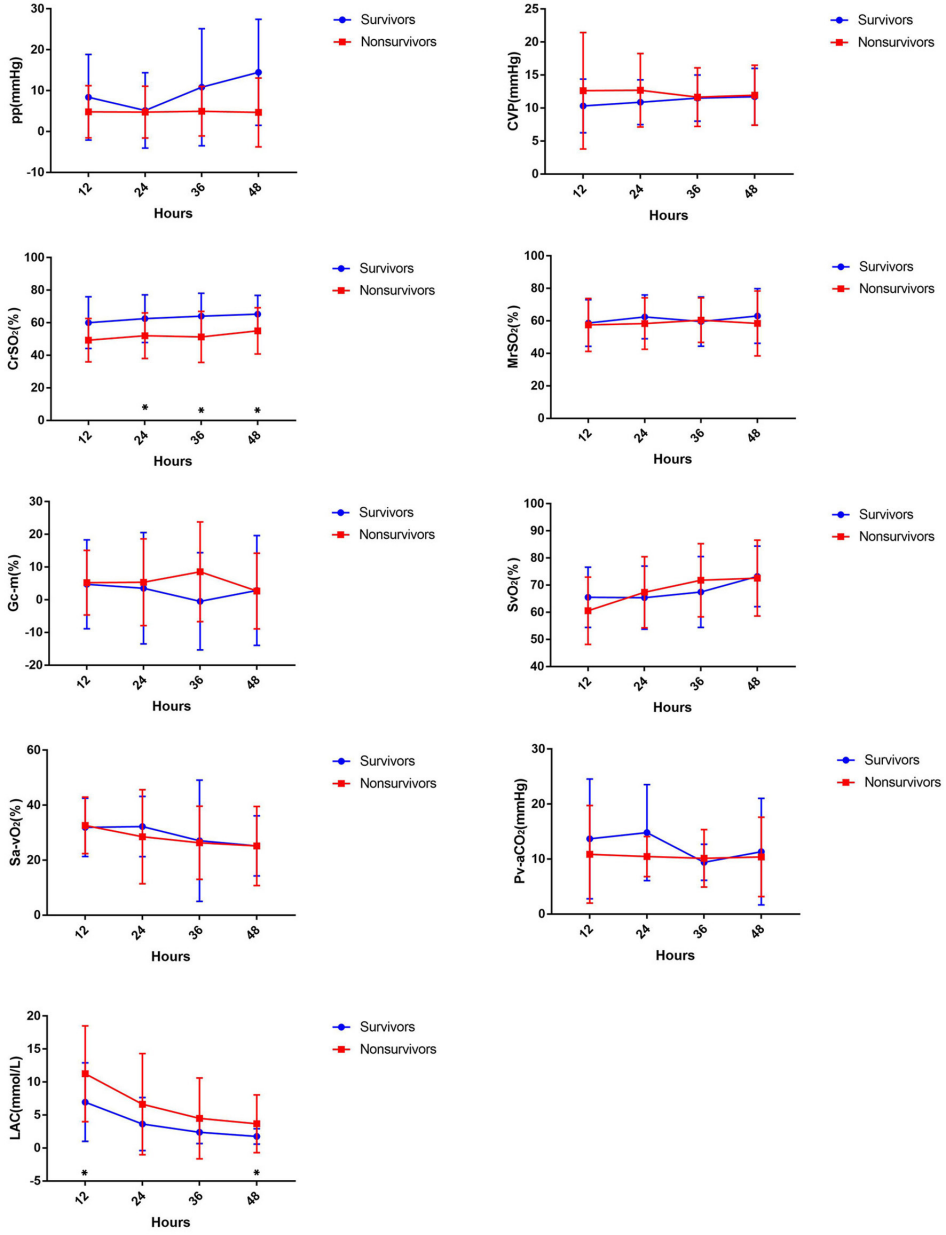
Comparing hemodynamic parameters in the first 48 h between survivors and non-survivors during ECMO.

The changes in monitoring indicators during ECMO are shown in Figure 1. Lactate levels were significantly higher in non-survivors compared to the survivors at 12 h (11.25 ± 7.26 vs. 6.96 ± 5.95 mmol/l, *p* = 0.022) and 48 h [2.2 (0.7, 20) vs. 1.4 (0.7, 5.8) mmol/l, *p* = 0.008]. The CrSO_2_ was significantly higher in survivors compared to non-survivors at 24 h (62.5 ± 14.61 vs. 52.05 ± 13.98%, *p* = 0.028), 36 h (64.04 ± 14.12 vs. 51.27 ± 15.65%, *p* = 0.005), and 48 h (65.32 ± 11.51 vs. 55.00 ± 14.18%, *p* = 0.008) (Figure 1 and Table 3).

**Table 3:** Comparison between the monitoring parameters of survivors and non-survivors.

	**Total (*N* = 55)**	**Survivors (*N* = 31)**	**Non-survivors (*N* = 24)**	***P*-value**
**ECMO-12 h**				
mABP (mmHg)	58.56 ± 15.59	59.00 ± 12.26	58.00 ± 19.34	0.816
CVP (mmHg)	11.38 ± 6.72	10.32 ± 4.06	12.63 ± 8.82	0.221
CrSO2 (%)	54.68 ± 15.38	60.07 ± 15.87	49.29 ± 13.31	0.062
MrSO2 (%)	58.11 ± 15.09	58.71 ± 14.34	57.50 ± 16.32	0.836
Gc-m (%)	5.93 (−18, 30)	5.51 (−18,30)	6 (−8.8,27)	0.927
SvO2 (%)	63.34 ± 11.71	65.51 ± 1.10	60.58 ± 12.40	0.306
Sa-vO2 (%)	32.24 ± 10.25	31.94 ± 10.61	32.63 ± 10.28	0.870
Pv-aCO2 (%)	12.44 ± 9.95	13.68 ± 10.89	10.87 ± 8.88	0.496
Lac (mmol/l)	8.74 ± 6.80	6.96 ± 5.95	11.25 ± 7.26	0.022^*^
**ECMO-24 h**				
mABP (mmHg)	59.98 ± 16.74	62.00 ± 14.99	57.38 ± 18.76	0.314
CVP (mmHg)	11.68 ± 4.52	10.88 ± 3.40	12.70 ± 5.55	0.152
CrSO2 (%)	57.41 ± 15.07	62.5 ± 14.61	52.05 ± 13.98	0.028^*^
MrSO2 (%)	60.49 ± 14.59	62.47 ± 13.46	58.39 ± 15.81	0.402
Gc-m (%)	2 (−0.26, 41)	−2 (−14, 41)	6 (−26, 24)	0.499
SvO2 (%)	66.16 ± 15.06	65.40 ± 11.61	67.37 ± 13.09	0.639
Sa-vO2 (%)	30.79 ± 13.57	32.23 ± 10.96	28.52 ± 17.11	0.432
Pv-aCO2 (%)	13.13 ± 7.44	14.46 ± 3.66	10.46 ± 3.66	0.047
Lac (mmol/l)	2.8 (0.8, 28)	2.3 (0.8, 19)	3.4 (0.9, 28)	0.107
**ECMO-36 h**				
mABP (mmHg)	63.17 ± 15.76	64.25 ± 14.62	61.26 ± 16.97	0.449
CVP (mmHg)	11.57 ± 3.90	11.51 ± 3.51	11.65 ± 4.44	0.897
CrSO2 (%)	58.06 ± 16.04	64.04 ± 14.12	51.27 ± 15.65	0.005^*^
MrSO2 (%)	60.00 ± 14.32	59.60 ± 15.11	60.48 ± 13.69	0.839
Gc-m (%)	67.6 (33, 95.4)	−3.1 (−22.2, 30.4)	8.25 (−20.2, 44.3)	0.097
SvO2 (%)	69.45 ± 13.28	67.48 ± 13.05	71.81 ± 13.49	0.288
Sa-vO2 (%)	26.72 ± 18.40	27.06 ± 22.04	26.32 ± 13.31	0.896
Pv-aCO2 (%)	9.74 ± 4.23	9.42 ± 3.27	10.13 ± 5.22	0.584
Lac (mmol/l)	3.28 ± 4.28	2.38 ± 1.71	4.49 ± 6.13	0.122
**ECMO-48 h**				
mABP (mmHg)	63.81 ± 12.32	66.07 ± 12.25	60.73 ± 12.01	0.124
CVP (mmHg)	11.82 ± 4.36	11.71 ± 4.30	11.95 ± 4.54	0.8445
CrSO2 (%)	60.61 ± 13.68	65.32 ± 11.51	55.00 ± 14.18	0.009^*^
MrSO2 (%)	61.00 ± 18.23	63.04 ± 16.84	58.45 ± 19.97	0.407
Gc-m (%)	6 (−25, 29)	2 (−25, 29)	6 (−19, 19)	0.987
SvO2 (%)	72.94 ± 12.28	73.20 ± 11.15	72.59 ± 13.99	0.871
Sa-vO2 (%)	25.18 ± 12.34	25.20 ± 10.92	25.15 ± 14.37	0.99
Pv-aCO2 (%)	10.94 ± 8.65	11.35 ± 9.68	10.39 ± 7.21	0.720
Lac (mmol/l)	1.7 (0.7, 20)	1.4 (0.7, 5.8)	2.2 (0.7, 20)	0.008^*^

*mABP, mean arterial blood pressure; CVP, central venous pressure; CrSO_2_, cranial regional oxygen saturation; MrSO_2_, mesenteric regional oxygen saturation; Gc-m, gap of CrSO_2_ and MrSO_2_(calculated by CrSO_2_ minus MrSO_2_); SvO_2_, mixed venous saturation; Sa-vO_2_, arteriovenous difference of partial pressure of oxygen; Pv-aCO_2_, venoarterial difference of partial pressure of carbon dioxide*.

* *p < 0.05*.

Multivariate logistics regression analysis for the hemodynamic and laboratory parameters revealed that the CrSO_2_ at 36 h (OR = 0.945, *p* = 0.049) and 48 h (OR = 0.919, *p* = 0.032) was related to mortality. Besides, CRRT (OR = 14.940, *p* = 0.039) was also related to mortality (Table 4).

**Table 4:** Multivariate logistics regression analysis of the hemodynamic monitoring indices during ECMO and their association with mortality.

	**OR**	**95%**	***P***
ECMO 12 h			
CrSO_2_ (%)	0.885	0.778–1.007	0.064
Lac (mmol/l)	0.969	0.763–1.230	0.795
CRRT	20.278	0.709–579.616	0.079
TΔp (h)	1.051	0.998–1.106	0.060
ECMO 24 h			
CrSO_2_ (%)	0.950	0.887–1.017	0.138
Lac (mmol/l)	1.068	0.850–1.341	0.572
CRRT	8.879	0.606–130.038	0.111
TΔp (h)	1.023	0.997–1.049	0.081
ECMO 36 h			
CrSO_2_ (%)	0.945	0.893–0.999	0.049^*^
Lac (mmol/l)	0.99	0.760–1.290	0.941
CRRT	10.687	0.890–128.329	0.062
TΔp (h)	1.016	0.994–1.038	0.149
ECMO 48 h			
CrSO_2_ (%)	0.919	0.850–0.993	0.032^*^
Lac (mmol/l)	1.056	0.564–1.977	0.864
CRRT	14.940	1.148–194.460	0.039^*^
TΔp (h)	1.011	0.990–1.034	0.302

*CrSO_2_, cranial regional oxygen saturation; CRRT, continuous renal replacement therapies; TΔp, time to achieve pulse pressure 10 mmHg*.

* *p < 0.05*.

**Figure 2:**
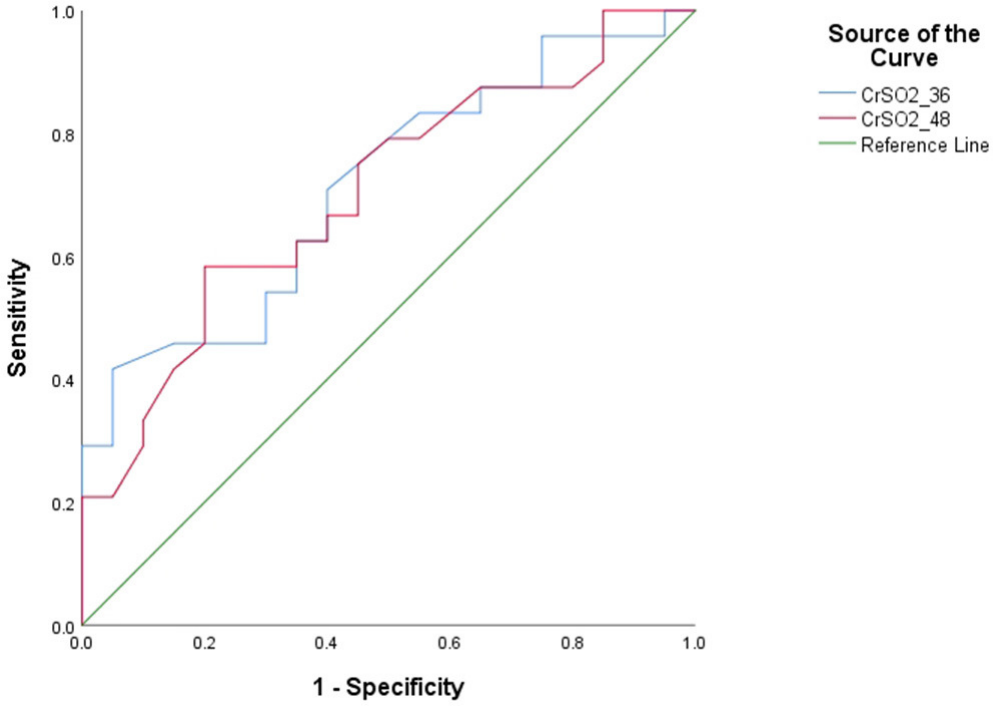
Receiver operating characteristic curves (ROC) of CrSO2 (36 h) and CrSO2 (48 h) between survivors and non-survivors.

The area under the ROC curve was 0.769 (*p* = 0.03) for CrSO_2_ at 36 h and 0.758 (*p* = 0.038) at 48 h (Figure 2). The optimal cutoff value for CrSO_2_ for the prediction of mortality after weaning off ECMO was 57% at 36 h (sensitivity: 61.5%, specificity: 80%) and 56% at 48 h (sensitivity: 76.9%, specificity: 70%) (Table 5).

**Table 5:** Cut-off of CrSO_2_ for the prediction of mortality.

	**Area under the curve**	***P*-value**	**Cutoff (%)**	**Sensitivity (%)**	**Specificity (%)**
CrSO_2_ (36 h)	0.769	0.030	57	61.5	80
CrSO_2_ (48 h)	0.758	0.038	56	76.9	70

*CrSO_2_, cranial regional oxygen saturation*.

**Figure 3:**
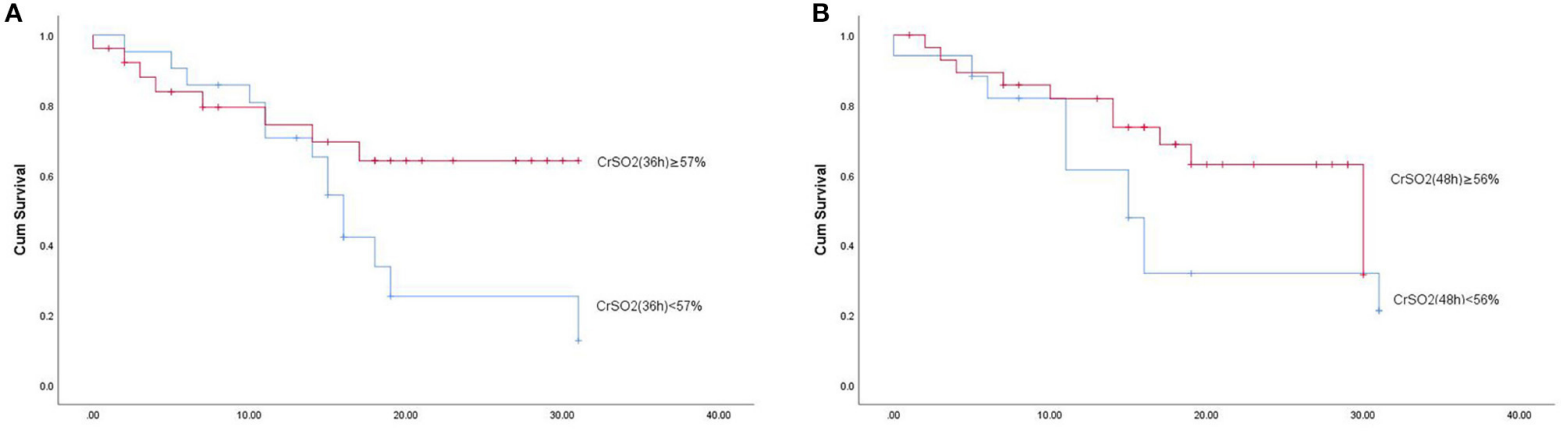
Kaplan-Meier analysis according to CrSO2 (36 h) and CrSO2 (48 h) between survivors and non-survivors.

The risk of mortality was higher among patients with a CrSO_2_(36h) <57% (*p* = 0.028) by Kaplan-Meier analysis. However, there was no difference in the mortality with CrSO_2_ (48 h) <56% (*p* = 0.103) (Figure 3).

## Discussion

Recent technological advancements have led to dramatic improvements in the prognosis of patients undergoing treatment with ECMO. In children, the assessment of organ perfusion using flow alone is limited due to differences in body weight and vascular volume. Therefore, enhanced tissue perfusion monitoring is essential during ECMO. However, no guidelines exist for the monitoring parameters for VA-ECMO.

Lactate, a routine ECMO monitoring indicator, was found to be associated with prognosis in this study. Park et al. (8) have reported that the appropriate cut-off values for predicting mortality were 7.05 mmol/L at 6 h, 4.95 mmol/L at 12 h, and 4.15 mmol/L at 24 h. Kim et al. (9) revealed that survivors had mean lactate levels of 3.85 mmol/L after the first day (vs. 10.69 mmol/L among non-survivors) and that the optimal cutoff value was 4.66 mmol/L (sensitivity: 75%, specificity: 75%). Our study also found a significant difference in the blood lactate level between survivors and non-survivors. In our study, lactate levels were significantly higher in non-survivors compared to survivors at 12 h (11.25 ± 7.26 vs. 6.96 ± 5.95 mmol/l, *p* = 0.022) and 48 h [2.2 (0.7, 20) vs. 1.4 (0.7, 5.8) mmol/l, *p* = 0.008], but no significant difference was observed on multivariate logistic regression, which meant that lactate was not a predictor of mortality.

There were no significant differences between the survival and non-survival groups in other routine monitoring measures, such as SvO_2_, Sa-vO_2_, and Pv-aCO_2_, which can be used to assess circulation state and discriminate shock. A study that investigated infants with CHD postoperatively showed that the sensitivity and specificity of Pv-aCO_2_ ≥ 12.3 mmHg in predicting oxygen supply/oxygen consumption ≤ 2 were 78.6 and 82.1%, respectively (10).

The area under the ROC curve was 0.769 (*p* = 0.03) for CrSO_2_ at 36 h and 0.758 (*p* = 0.038) at 48 h. The optimal cutoff values for CrSO_2_ for the prediction of mortality after weaning off ECMO was 57% at 36 h (sensitivity: 61.5%, specificity: 80%) and 56% at 48 h (sensitivity: 76.9%, specificity: 70%). Similarly, Tsou et al. (11) showed that any regional oxygen saturation index (rSO_2_) levels ≤ 50% were associated with unfavorable outcomes at hospital discharge [multivariable-adjusted odds ratio (OR), 2.82 (95% CI: 1.10–7.25)]. Kim et al. also showed the optimal cutoff values for right-sided and left-sided CrSO_2_ for predicting mortality were 58% (sensitivity: 78.7%, specificity: 83.3%) and 57% (sensitivity: 80.0%, specificity: 70.8%), respectively (9).

Previous studies have found that the differences in cerebral and abdominal oxygen possess a certain clinical significance in assessing perfusion (12–14). Generally speaking, abdominal oxygen varied in a wide range and decreased when cardiac output was reduced. Therefore, the larger the difference between cerebral and abdominal oxygen is, the poorer the prognosis is. We compared the MrSO_2_ between the survival and non-survival groups. However, there was no significant difference in the MrSO_2_ or the gap between CrSO_2_ and MrSO_2._ This may be related to the variation in abdominal oxygen in children, which is susceptible to the influence of abdominal bloating, peritoneal dialysis, and urine retention.

Similarly, our study showed that CRRT (OR = 14.940, *p* = 0.039) was related to mortality, as reported by some previous studies (15–18). Acute kidney injury (AKI) was a common occurrence in patients receiving ECMO after congenital heart surgery, and some studies have demonstrated an association between AKI and mortality (15, 16). Pilar et al. (17) found that the use of CRRT during ECMO was associated with higher mortality (OR: 6.12, *p* = 0.06). A systematic review conducted by Chen et al. (18) also showed higher mortality (OR: 5.89, *p* < 0.0001) and longer ECMO duration in patients requiring CRRT while on ECMO support. However, the drawback of the study was that kidney regional saturation was not monitored by NIRS due to the difficulty of the electrode adhesion to the costovertebral angle for children implanted ECMO in a central cannulation way.

Our study has some other limitations. First, statistical bias was inevitable owing to the retrospective design of the study. Further study should be applied in a proper prospective randomized way. Second, the changes in brain oxygen caused by brain injury were not excluded using computed tomography or magnetic resonance imaging. Finally, the study focused more on monitoring during ECMO and failed to analyze the data before and after ECMO initiation.

In conclusion, cerebral oxygen monitoring has important clinical significance as a non-invasive real-time monitoring technique for assessing perfusion and prognosis. Therefore, NIRS may be instrumental for monitoring the hemodynamic stability during the early period of ECMO, and cranial regional oxygen saturation (CrSO_2_) could predict hospital mortality after weaning patients off ECMO. However, more studies should be performed to validate the clinical significance of NIRS in children with ECMO after cardiac surgery.

## Data Availability Statement

The original contributions presented in the study are included in the article/supplementary material, further inquiries can be directed to the corresponding authors.

## Author Contributions

MZ and XC: statistics and drafting of the article. YY and YS: data collection. LZ and XG: data interpretation. ZX and HZ: concept and design. All authors contributed to the article and approved the submitted version.

## Conflict of Interest

The authors declare that the research was conducted in the absence of any commercial or financial relationships that could be construed as a potential conflict of interest.

## Publisher's Note

All claims expressed in this article are solely those of the authors and do not necessarily represent those of their affiliated organizations, or those of the publisher, the editors and the reviewers. Any product that may be evaluated in this article, or claim that may be made by its manufacturer, is not guaranteed or endorsed by the publisher.
